# MOCAT: A Metagenomics Assembly and Gene Prediction Toolkit

**DOI:** 10.1371/journal.pone.0047656

**Published:** 2012-10-17

**Authors:** Jens Roat Kultima, Shinichi Sunagawa, Junhua Li, Weineng Chen, Hua Chen, Daniel R. Mende, Manimozhiyan Arumugam, Qi Pan, Binghang Liu, Junjie Qin, Jun Wang, Peer Bork

**Affiliations:** 1 Structural and Computational Biology Unit, European Molecular Biology Laboratory, Heidelberg, Germany; 2 Department of Science and Technology, BGI-Shenzhen, Shenzhen, Guangdong, China; 3 School of Bioscience and Biotechnology, South China University of Technology, Guangzhou, Guangdong, China; 4 Max-Delbruck-Centre for Molecular Medicine, Berlin-Buch, Germany; Argonne National Laboratory, United States of America

## Abstract

MOCAT is a highly configurable, modular pipeline for fast, standardized processing of single or paired-end sequencing data generated by the Illumina platform. The pipeline uses state-of-the-art programs to quality control, map, and assemble reads from metagenomic samples sequenced at a depth of several billion base pairs, and predict protein-coding genes on assembled metagenomes. Mapping against reference databases allows for read extraction or removal, as well as abundance calculations. Relevant statistics for each processing step can be summarized into multi-sheet Excel documents and queryable SQL databases. MOCAT runs on UNIX machines and integrates seamlessly with the SGE and PBS queuing systems, commonly used to process large datasets. The open source code and modular architecture allow users to modify or exchange the programs that are utilized in the various processing steps. Individual processing steps and parameters were benchmarked and tested on artificial, real, and simulated metagenomes resulting in an improvement of selected quality metrics. MOCAT can be freely downloaded at http://www.bork.embl.de/mocat/.

## Introduction

The emerging field of metagenomics has enabled researchers to study the structure, dynamics, and functionality of uncultured microbial communities. Processing the vast amounts of metagenomics data usually involves quality-controlling raw sequence reads, aligning them to reference databases, and assembling them into longer contigs prior to predicting genes. Several packages are available for processing and analyzing metagenomics data, either as web- and cloud-based services or stand-alone computational pipelines [1]–[7]. But currently none of them supports the assembly and gene prediction of metagenomics data produced by the Illumina platform.

As exemplified by recent clinical, large-scale, and on-going studies (e.g., the Human and Earth Microbiome Projects), the usage of high throughput sequencing (HTS) data can be anticipated to further increase considerably in both terms of data volume and scope of application [8]–[11]. Thus, there is an imminent need for applications providing standardized methods for processing of HTS data in the form of pipelines [12] to facilitate comparative downstream analyses.

To address these issues, we have developed MOCAT, a metagenomics assembly and gene prediction toolkit for both small and large-scale processing of metagenomic data produced by the Illumina sequencing technology.

## Results and Discussion

**Figure 1:**
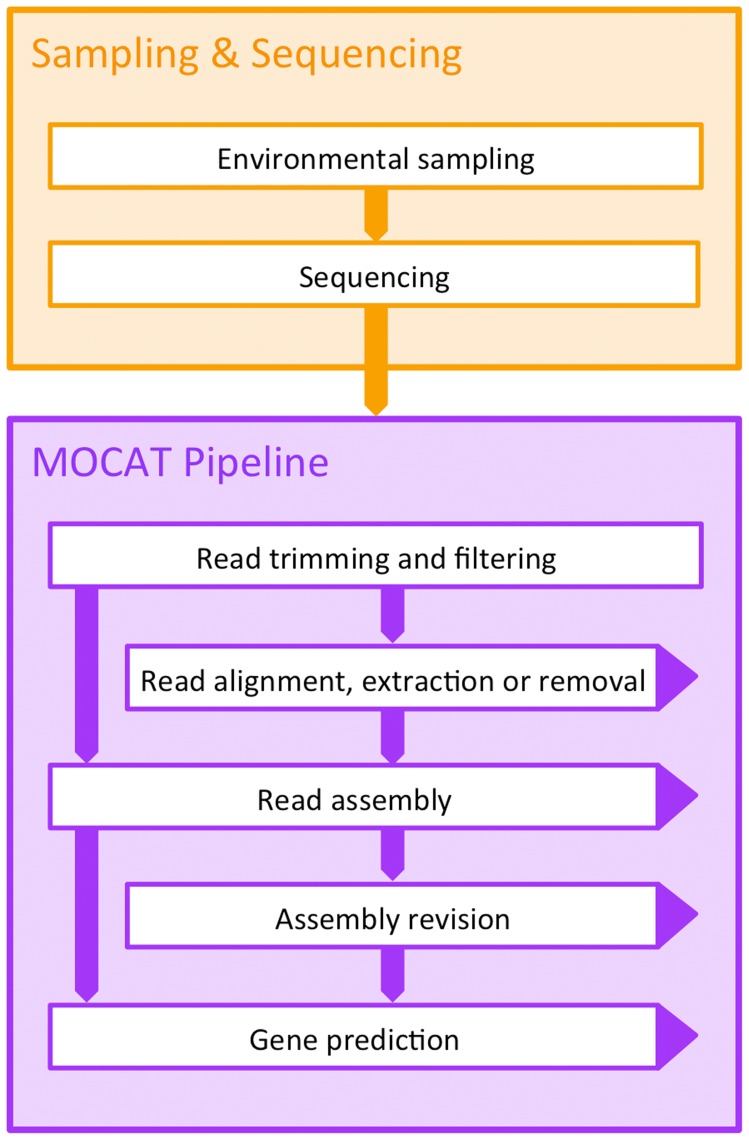
The MOCAT data processing pipeline. Metagenomic samples are collected and sequenced. The raw sequence reads are given as input to the pipeline, which are processed by modular steps resulting in metagenome assemblies and predicted genes. Arrows extending to the right from boxes, indicate input to various downstream analyses. Statistics from each step are summarized into multi-sheet Excel documents, as well as queryable SQLite databases.

The main pipeline is divided into five major steps: (i) quality trimming and filtering of raw reads, (ii) optional mapping to remove, extract, and/or quantify reads matching a reference database, (iii) assembly, (iv) assembly revision, and (v) gene prediction (Figure 1). Statistics from each step are summarized into multi-sheet Excel documents, as well as queryable SQLite databases. Full details of output files and statistics produced in each processing step are given in Table S7.

The individual processing steps in MOCAT were benchmarked using three different data sets: 124 published human gut metagenomic samples [8], a mock community produced by the Human Microbiome Project (HMP) with 22 species from 19 genera [9], and a simulated metagenome with 100 strains from 85 species [13]. By using this combination of host associated, artificial, and simulated metagenomes with different taxonomical resolution, we show that MOCAT can efficiently process a variety of metagenomic samples, ranging in both size (0.5–16.6 Gbp), origin and composition owing to new developments in each of the five major steps.

### i) Quality Trimming and Filtering of Raw Reads

Read quality trimming and filtering can greatly improve the length and accuracy of metagenomic assemblies [13]. Therefore, in the first processing step, raw reads below specified quality and length cutoffs are trimmed or removed using either the FastX program (http://hannonlab.cshl.edu/fastx_toolkit/) or the DynamicTrim algorithm in the SolexaQA package [14]. The supported FastX program removes bases from the 3′ end below a user-defined threshold, whereas the DynamicTrim algorithm in the SolexaQA package keeps the longest contiguous read segment in which all quality scores are above the user-defined threshold [14]. After quality trimming and filtering our three test datasets, 57–79% of the reads remained as high quality reads (Table S1).

Additionally, to reduce base composition-biases that commonly occur in HTS data [15], the frequency of each base at each position over all reads is calculated, and bases that exceed two standard deviations of the average base frequency within a sample are trimmed from the 5′ end of all reads. Using our test data set of 124 published human gut microbial samples, on average, the fraction of reads that could be mapped to assemblies was 1% higher when using 5′ trimmed reads, compared to non-trimmed reads (Table S2).

MOCAT also supports the FastQC package, for evaluating raw read quality statistics (http://www.bioinformatics.bbsrc.ac.uk/projects/fastqc).

### ii) Mapping, and Removal or Extraction of Reads Matching a Reference Database

In the second step, reads can be mapped to reference sequences in order to extract or remove reads from the original data set as well as to calculate base or read coverages. For example, reads from a human fecal metagenomic sample can be mapped to the provided human genome database (hg19, Genome Reference Consortium Human Reference 37) to remove reads of human origin using SOAPAligner2 [16], or reads containing adapters used for sequencing library construction can be removed using Usearch [17]. Reads can also be mapped to any other custom reference database to calculate base and insert coverage of reference sequences to estimate taxonomic and/or functional composition of a sample, for example.

**Figure 2:**
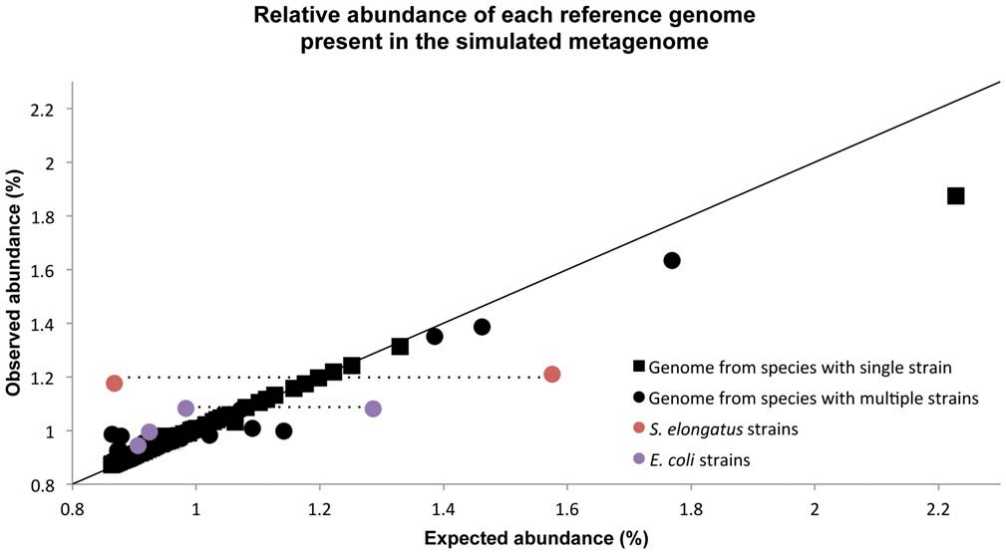
Relative abundance of each reference genome present in the simulated metagenome. The observed abundances by mapping reads to reference genomes and the expected abundance correlate with a Pearson correlation coefficient of 0.95 (base and read counts). Circles represent genomes with multiple strains from one species and squares represent genomes with only one strain within the species. All, but one, of the observations deviating from the diagonal are strains from the same species. These strains are either over- or under represented because reads are mapped to other closely related strains in addition to the strain of origin. Highlighted by dashed lines, are two examples where a high sequence similarity between strains (99.9% and 98.7% for the *Synechococcus elongatus* and *Escherichia coli* strains, respectively) can result in deviations from expected abundances.

Here, we estimated the taxonomic composition of the simulated metagenome by mapping reads to the set of original reference genomes (Table S2 in [13] and Table S3) and calculating genome size-normalized base and read coverages. The Pearson and Spearman correlation coefficients between the observed and expected composition of the simulated metagenome were 0.95 and 0.90, respectively, for both base and read counts (Figure 2), and only 80 out of more than 30 Million reads were not aligned. However, the observed abundances of genomes with very high sequence identity may deviate from the expected abundances due to reads mapping to both the genome of origin and other highly similar genomes.

**Figure 3:**
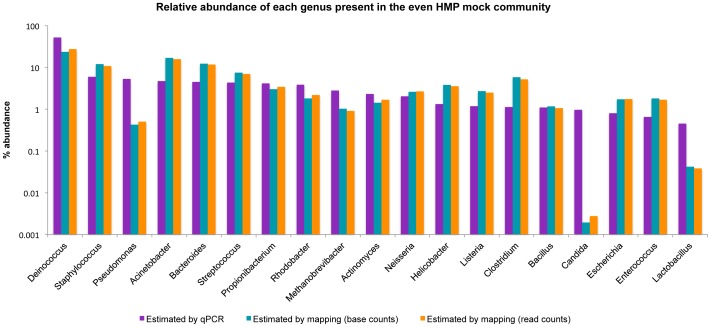
Relative abundance of each genus present in the even HMP mock community. The estimated abundances using qPCR and by mapping reads to reference genomes correlate with a Pearson correlation coefficient of 0.75 (base counts) and 0.83 (read counts).

When estimating taxonomic composition of the HMP mock community, reads were mapped to reference sequences of the community (Table S4). By first removing quality filtered and trimmed reads matching known Illumina adapter sequences (Table S5), the percentage of bases and reads mapping to the reference genomes increased from 94.3% to 97.3%, and 95.0% to 97.6%, respectively, indicating the usefulness of a pre-screening step. The taxonomic composition estimated here is similar to the values calculated by the HMP consortium (Pearson correlation coefficient of 0.75 and 0.83 for bases and reads mapping, respectively, Figure 3), and also to estimates of 16S sequences using 454 sequencing presented in (Figure S14, [18]). Experimental errors, not applicable to estimates of computationally simulated metagenomes, may explain the lower correlation in the mock community, compared to the simulated metagenome.

### iii) Assembly

In the assembly step, a new version (1.06) of SOAPdenovo [19] is used. For paired-end sequences, the insert size of each sequencing library is estimated at run-time by mapping reads to either reference marker genes [20] prior to assembly, or assembled contigs prior to scaffolding. Similarly, Kmer sizes used for assemblies are calculated at run-time for each individual metagenome. Empirical tests on a large number of samples show that estimating a Kmer size for each sample as the closest odd number larger or equal to half the average read length may not yield the best possible assembly, but balances assembly throughput and accuracy.

The accuracy of metagenomic assemblies was assessed using data from the simulated metagenome and the mock community. We used the percentage of predicted complete genes aligning to the reference sequences of origin, as a proxy for correctly assembled scaftigs (contigs that were extended and linked using the paired-end information of sequencing reads). For the simulated metagenome this value was 95.2% (12,385 complete genes predicted), and for the mock community 89.3% of the complete genes aligned (1,042 complete genes predicted). The lower number of predicted complete genes in the mock community may be explained by the relatively low number of high quality reads used in the assembly for this metagenome.

The effect of using variable Kmer sizes, rather than a fixed kmer, in the assembly step, was evaluated using the 124 gut metagenomes. Estimating Kmer sizes at run-time for each individual metagenome, rather than using a fixed Kmer size across all samples, improved the number and frequency of complete gene calls as well as overall average gene length (column 1 in Table 1).

**Table 1:** Progressive improvement of gene prediction metrics in 124 human gut metagenomes.

Quality metric	Improvement compared to fixed kmer = 23 (%)	
	No assembly revision	Revised assembly
Number of complete genes	8.1	10.2
Number of complete genes/Mbp	4.6	18.5
Average gene length	1.7	1.8

Gene prediction metrics are improved when using an automated kmer size in SOAPdenovo and with assembly revision (correction of base errors, short indels, and chimeric contigs), compared to a fixed kmer size of = 23 in SOAPdenovo and no assembly revision. The Kmer size is estimated as the closest odd number greater than half the average read length for a sample. Numbers reported are in percent improvement of the respective quality metric. The calculated Kmer for each sample is given in Table S8.

### iv) Assembly Revision

In the assembly revision step, a feature independent of the utilized assembly packages, MOCAT can revise existing paired-end read assemblies by aligning the reads to assembled scaftigs using the gap-tolerant BWA aligner [21] to correct for base errors and short indels, and the fast SOAPaligner2 to resolve chimeric regions. Performing assembly revision on the 124 human fecal metagenomes further improved gene prediction metrics (column 2 in Table 1).

### v) Gene Prediction

Finally, protein coding genes on the metagenomes are predicted using either the default component Prodigal [22] or MetaGeneMark [23]. An in depth comparison of the gene prediction software is beyond the scope of this article. However, each software have been benchmarked by the respective authors (http://prodigal.ornl.gov/results.php and [23]). An independent comparison determined that MetaGeneMark had a higher precision and Prodigal a higher recall rate (http://genome.jgi.doe.gov/programs/metagenomes/benchmarks.jsf).

### Conclusions

The functionality and versatility of the pipeline has been demonstrated using an artificial mock community metagenome, a simulated metagenome with 100 species, and 124 human gut metagenomes. Based on parameter exploration and data driven parameter optimization at run-time, the MOCAT pipeline can process metagenomes in a standardized and automated way while improving the quality of assembly and gene prediction compared to using default parameters for the supported programs. To date, MOCAT has additionally been used to process and assemble hundreds of host-associated and ocean metagenomes within the scope of the MetaHIT [8] and TARA Oceans projects [24].

### Implementation, Availability, and Requirements

MOCAT is implemented in Perl and installed by extracting the package and executing one script, which downloads the default external software used by the pipeline and sets up the software. This reduces the otherwise tedious process of downloading all the individual components, a common drawback of in-house pipelines [12]. Optional components requiring a license, such as MetaGeneMark [23] for gene prediction, and Usearch [17] for removal or extraction of reads by alignment to a FASTA-formatted sequence file, require a manual download.

A new project is quickly setup requiring only single- or paired-end FastQ formatted sequencing reads files [25] for each sample in a separate directory. The use of a project-specific configuration file, with suggested default settings, offers users to run all processing steps up to gene prediction without additional setup, while allowing experienced users to modify parameters and programs used in MOCAT. All of the settings are described in the MOCAT documentation.

A queuing system enables processing of a large number of samples in parallel. If present, MOCAT seamlessly integrates all processing steps with the SGE and PBS queuing systems. However, if no queuing system is available, MOCAT processes samples serially on the machine it was executed.

MOCAT runs on 64-bit UNIX systems and can be freely downloaded at http://www.bork.embl.de/mocat/. Perl version 5.8.8 or above is required. MOCAT is also available in a Virtual Machine package, which could be used to run MOCAT on a PC or a cloud based system. The open source code and modular architecture allow users to modify or exchange the programs that are utilized in the various processing steps. There are no minimum hardware requirements for the pipeline itself to run, however, requirements for analyzing metagenomic datasets vary depending on the number of samples to process in parallel and the sequencing depth of each sample. To aid in determining whether local computational resources are adequate, we provide in Table S6 and S8 the maximum resources required to process the datasets in this article. We recommend at least 16 GB of RAM to process smaller metagenomes and 64 GB of RAM to process medium sized metagenomes, but these requirements may vary depending on project settings and systems used. The hard disk space requirements depend on the size and number of metagenomes to analyze, but we recommend at least 500 GB of hard disk space.

## Methods

### Data Sources

Data for the simulated metagenome is publically available at http://www.bork.embl.de/~mende/simulated_data/ [13]. This dataset consisted of simulated paired-end raw reads and 193 reference sequences (chromosomes and plasmids) from 100 genomes used to simulate this metagenome (Table S3). Metagenomic data for the even HMP mock community were downloaded from http://www.ncbi.nlm.nih.gov/bioproject/48475, and the references sequences were downloaded from the NCBI database (Table S4), with the exception of *Candida albicans*, which was downloaded from http://www.candidagenome.org/download/sequence/C_albicans_SC5314/Assembly21/current/. Metadata for the mock community was downloaded from http://www.hmpdacc.org/HMMC/. Datasets for the simulated metagenome and the mock community can optionally be downloaded automatically when installing the MOCAT pipeline.

Raw reads for the 124 human gut microbiomes were downloaded from the EBI homepage (accession number ERA000116, http://ftp.sra.ebi.ac.uk/vol1/ERA000/ERA000116/fastq/).

### Data Processing and Software Settings

The three datasets were processed by the *read_trim_filter* step in MOCAT with length cut off set to *30* and quality cut off set to *20*, using *solexaqa* for the mock community and the simulated metagenome, and *fastx* for the 124 gut metagenomes.

Estimated taxonomic compositions for the simulated metagenome and the mock community were calculated in three steps. First, quality trimmed and filtered reads from the mock community were screened against a FASTA-file with Illumina adapter sequences (Table S5), using the *screen_fastafile* option and e-value set to *0.01*. Second, screened reads from the mock community and quality trimmed and filtered reads from the simulated metagenome were mapped and filtered against the custom-made reference databases with chromosome and plasmid sequences from the 22 mock genomes (Table S4) and 100 genomes from the simulated metagenome (Table S2 in [13] and Table S3), respectively. This was done by executing the *screen* and *filter* commands with length cutoff set to *30*, percentage identity set to *90 and* paired_end_filtering set to *yes* for the simulated metagenome and set to *no* for the mock community. Finally, the taxonomic composition was estimated using the *calculate_coverage* command.

Assembly and gene prediction, on the simulated metagenome and mock community, were performed using the *assembly* (SOAPdenovo version *1.06*) and *gene_prediction* (*MetaGeneMark*) options. Quality trimmed and filtered reads from the simulated metagenome, and adapter-screened reads from the mock community, were assembled into scaftigs *60* bp or longer. Predicted complete genes were aligned to their respective metagenomes using blastall v2.2.26 [26] (program blastn, 95% sequence identity, alignment length > = 90%, and e-value 0.1) and only the best hit selected.

The 124 human gut microbiomes were processed with and without 5′ trimming. 5′ trimmed reads were assembled using SOAPdenovo *1.05*, using both the Kmer determined by MOCAT and a fixed Kmer size set to 23. These assemblies were revised using SOAPdenovo *1.06* using the *assembly_revision* options, and genes were predicted, with *MetaGeneMark* as selected software, on scaftigs from both assemblies and revised assemblies. The non 5′ trimmed and 5′ trimmed reads were mapped to the assembled scaftigs using the *screen* option using length cutoff *30* and quality cutoff *15*.

Complete commands for processing the simulated metagenome and mock community in MOCAT are bundled with the installation of the pipeline.

## Supporting Information

Table S1 **Raw and high quality read and base statistics for the three metagenomic data sets used in this study.**
(DOCX)

Table S2 **Comparison of mapping rates of 5′ untrimmed and 5′ trimmed reads.**
(DOCX)

Table S3 **Mapping used when summarizing the estimated abundances for the simulated metagenome.**
(DOC)

Table S4 **Reference sequences to which reads from the even HMP mock community were mapped.**
(DOCX)

Table S5 **Aligned raw reads form the mock community to known Illumina adapters.**
(DOCX)

Table S6 **Maximum computational resources and processing time required for each processing step, for each of the datasets used in this article.**
(DOC)

Table S7 **Output files and statistics from each of the processing steps in MOCAT.**
(DOCX)

Table S8 **Number of raw and high quality (HQ) bases and reads, calculated Kmer size, and the computational resources (RAM and HDD) required to assemble the 124 fecal metagenomics samples.**
(DOCX)
