# Supplementary material for: MOCAT: A Metagenomics Assembly and Gene Prediction Toolkit
Source: PLoS One. 2012 Oct 17;7(10):e47656. doi: 10.1371/journal.pone.0047656 (PMC3474746; doi:10.1371/journal.pone.0047656)
Supplement: Table S1 — Raw and high quality read and base statistics for the three metagenomic data sets used in this study. (DOCX) [file pone.0047656.s001.docx]

**Table S1.** Raw and high quality read and base statistics for the three metagenomic data sets used in this study.

| **Metagenome** | **Raw reads** | **Raw bases** | **High quality reads** | **High quality bases** |
| --- | --- | --- | --- | --- |
| Simulated metagenome | 53,334,008 | 4,000,050,600 | 30,427,779 | 1,503,286,668 |
| Mock community | 6,562,065 | 492,154,875 | 3,943,759 | 206,197,036 |
| 124 gut metagenomes (average) | 45,411,302 | 3,239,648,242 | 35,789,998 | 2,049,553,760 |
