# Supplementary material for: MOCAT: A Metagenomics Assembly and Gene Prediction Toolkit
Source: PLoS One. 2012 Oct 17;7(10):e47656. doi: 10.1371/journal.pone.0047656 (PMC3474746; doi:10.1371/journal.pone.0047656)
Supplement: Table S2 — Comparison of mapping rates of 5′ untrimmed and 5′ trimmed reads. (DOCX) [file pone.0047656.s002.docx]

**Table S2.** Comparison of mapping rates of 5’ untrimmed and 5’ trimmed reads. Raw data from 124 fecal metagenomes [8] were quality processed by not trimming and trimming the 5’ end of reads. The fraction of reads mapping to contigs was 1% higher for the 5’ trimmed reads.

|  | **5'-untrimmed** | **5'-trimmed** |
| --- | --- | --- |
| Total number of reads (Mio.) | 4,510 | 4,377 |
| **2 mismatches allowed** |  |  |
| Number of mapped reads (Mio.) | 2,398 (53.2%) | 2,363 (54.0%) |
| **0 mismatches allowed** |  |  |
| Number of mapped reads (Mio.) | 1,889 (41.9%) | 1,876 (42.9%) |
