# Supplementary material for: MOCAT: A Metagenomics Assembly and Gene Prediction Toolkit
Source: PLoS One. 2012 Oct 17;7(10):e47656. doi: 10.1371/journal.pone.0047656 (PMC3474746; doi:10.1371/journal.pone.0047656)
Supplement: Table S3 — Mapping used when summarizing the estimated abundances for the simulated metagenome. (DOC) [file pone.0047656.s003.doc]

**Table S3.** Mapping used when summarizing the estimated abundances for the simulated metagenome. The estimated abundance for each of the 100 species was calculated by summarizing the estimated abundance of the reference sequences.

| **Species** | **References** |
| --- | --- |
| Escherichia coli str. K12 substr. W3110 | AC_000091 |
| Haemophilus influenzae Rd KW20 | NC_000907 |
| Escherichia coli str. K12 substr. MG1655 | NC_000913 |
| Chlamydophila pneumoniae CWL029 | NC_000922 |
| Rickettsia prowazekii str. Madrid E | NC_000963 |
| Bacillus subtilis subsp. subtilis str. 168 | NC_000964 |
| Ureaplasma parvum serovar 3 str. ATCC 700970 | NC_002162 |
| Bacillus halodurans C-125 | NC_002570 |
| Lactococcus lactis subsp. lactis Il1403 | NC_002662 |
| Bordetella bronchiseptica RB50 | NC_002927 |
| Geobacter sulfurreducens PCA | NC_002939 |
| Mycobacterium avium subsp. paratuberculosis K-10 | NC_002944 |
| Mycobacterium bovis AF2122/97 | NC_002945 |
| Neisseria gonorrhoeae FA 1090 | NC_002946 |
| Staphylococcus aureus subsp. aureus MRSA252 | NC_002952 |
| Treponema denticola ATCC 35405 | NC_002967 |
| Coxiella burnetii RSA 493 | NC_002971,NC_004704 |
| Sinorhizobium meliloti 1021 | NC_003047,NC_003037,NC_003078 |
| Streptococcus pneumoniae R6 | NC_003098 |
| Neisseria meningitidis MC58 | NC_003112 |
| Yersinia pestis CO92 | NC_003143,NC_003131,NC_003132 |
| Chlamydophila caviae GPIC | NC_003361,NC_004720 |
| Bacillus cereus ATCC 10987 | NC_003909,NC_005707 |
| Staphylococcus aureus subsp. aureus MW2 | NC_003923 |
| Bacillus anthracis str. Ames | NC_003997 |
| Streptococcus pyogenes MGAS315 | NC_004070 |
| Streptococcus agalactiae 2603V/R | NC_004116 |
| Wigglesworthia glossinidia endosymbiont of Glossina brevipalpis | NC_004344,NC_003425 |
| Corynebacterium efficiens YS-314 | NC_004369 |
| Bacteroides thetaiotaomicron VPI-5482 | NC_004663,NC_004703 |
| Bacillus cereus ATCC 14579 | NC_004722,NC_004721 |
| Prochlorococcus marinus str. MIT 9313 | NC_005071 |
| Chromobacterium violaceum ATCC 12472 | NC_005085 |
| Ralstonia eutropha H16 | NC_005241 |
| Rhodopseudomonas palustris CGA009 | NC_005296,NC_005297 |
| Borrelia garinii PBi | NC_006156,NC_006128,NC_006129 |
| Bacillus cereus E33L | NC_006274,NC_007103,NC_007104,NC_007105,NC_007106,NC_007107 |
| Thermus thermophilus HB8 | NC_006461,NC_006462,NC_006463 |
| Idiomarina loihiensis L2TR | NC_006512 |
| Synechococcus elongatus PCC 6301 | NC_006576 |
| Bacillus clausii KSM-K16 | NC_006582 |
| Candidatus Blochmannia pennsylvanicus str. BPEN | NC_007292 |
| Streptococcus pyogenes MGAS5005 | NC_007297 |
| Thiobacillus denitrificans ATCC 25259 | NC_007404 |
| Anabaena variabilis ATCC 29413 | NC_007413,NC_007410,NC_007411,NC_007412 |
| Pelodictyon luteolum DSM 273 | NC_007512 |
| Synechococcus elongatus PCC 7942 | NC_007604,NC_007595 |
| Sodalis glossinidius str. 'morsitans' | NC_007712,NC_007713,NC_007714,NC_007715 |
| Staphylococcus aureus subsp. aureus NCTC 8325 | NC_007795 |
| Lactobacillus salivarius UCC118 | NC_007929,NC_006529,NC_006530,NC_007930 |
| Psychrobacter cryohalolentis K5 | NC_007969,NC_007968 |
| Lawsonia intracellularis PHE/MN10 | NC_008011,NC_008012,NC_008013,NC_008014 |
| Haloquadratum walsbyi DSM 16790 | NC_008212,NC_008213 |
| Francisella tularensis subsp. tularensis FSC198 | NC_008245 |
| Cytophaga hutchinsonii ATCC 33406 | NC_008255 |
| Rhodococcus sp. RHA1 | NC_008268,NC_008269,NC_008270,NC_008271 |
| Haemophilus somnus 129PT | NC_008309,NC_006298 |
| Shewanella sp. MR-7 | NC_008322,NC_008320 |
| Alkalilimnicola ehrlichei MLHE-1 | NC_008340 |
| Syntrophomonas wolfei subsp. wolfei str. Goettingen | NC_008346 |
| Hyphomonas neptunium ATCC 15444 | NC_008358 |
| Rhodopseudomonas palustris BisA53 | NC_008435 |
| Leptospira borgpetersenii serovar Hardjo-bovis JB197 | NC_008510,NC_008511 |
| Buchnera aphidicola str. Cc (Cinara cedri) | NC_008513 |
| Lactococcus lactis subsp. cremoris SK11 | NC_008527,NC_008503,NC_008504,NC_008505,NC_008506,NC_008507 |
| Lactobacillus delbrueckii subsp. bulgaricus ATCC BAA-365 | NC_008529 |
| Listeria welshimeri serovar 6b str. SLCC5334 | NC_008555 |
| Escherichia coli APEC O1 | NC_008563,NC_009837,NC_009838 |
| Clostridium novyi NT | NC_008593 |
| Bacillus thuringiensis str. Al Hakam | NC_008600,NC_008598 |
| Psychromonas ingrahamii 37 | NC_008709 |
| Prochlorococcus marinus str. NATL1A | NC_008819 |
| Clostridium thermocellum ATCC 27405 | NC_009012 |
| Burkholderia pseudomallei 668 | NC_009074,NC_009075 |
| Mycobacterium sp. JLS | NC_009077 |
| Pseudomonas stutzeri A1501 | NC_009434 |
| Mycobacterium tuberculosis F11 | NC_009565 |
| Thermosipho melanesiensis BI429 | NC_009616 |
| Clostridium beijerinckii NCIMB 8052 | NC_009617 |
| Staphylococcus aureus subsp. aureus JH1 | NC_009632,NC_009619 |
| Alkaliphilus metalliredigens QYMF | NC_009633 |
| Methanococcus maripaludis C7 | NC_009637 |
| Staphylococcus aureus subsp. aureus str. Newman | NC_009641 |
| Ochrobactrum anthropi ATCC 49188 | NC_009667,NC_009668,NC_009669,NC_009670,NC_009671,NC_009672 |
| Ignicoccus hospitalis KIN4/I | NC_009776 |
| Staphylococcus aureus subsp. aureus Mu3 | NC_009782 |
| Escherichia coli E24377A | NC_009801,NC_009786,NC_009787,NC_009788,NC_009789,NC_009790,NC_009791 |
| Campylobacter concisus 13826 | NC_009802,NC_009795,NC_009796 |
| Herpetosiphon aurantiacus ATCC 23779 | NC_009972,NC_009973,NC_009974 |
| Salmonella enterica subsp. enterica serovar Paratyphi B str. SPB7 | NC_010102 |
| Chlamydia trachomatis L2b/UCH-1/proctitis | NC_010280 |
| Acinetobacter baumannii SDF | NC_010400,NC_010395,NC_010396,NC_010398,NC_010401,NC_010402,NC_010403,NC_010404 |
| Pseudomonas putida W619 | NC_010501 |
| Clostridium botulinum B1 str. Okra | NC_010516,NC_010379 |
| Corynebacterium urealyticum DSM 7109 | NC_010545 |
| Cyanothece sp. ATCC 51142 | NC_010546,NC_010547,NC_010539,NC_010541,NC_010542,NC_010543 |
| Mycobacterium marinum M | NC_010612,NC_010604 |
| Burkholderia phymatum STM815 | NC_010622,NC_010623,NC_010625,NC_010627 |
| Nostoc punctiforme PCC 73102 | NC_010628,NC_010629,NC_010630,NC_010631,NC_010632,NC_010633 |
| Erwinia tasmaniensis | NC_010694,NC_010693,NC_010695,NC_010696,NC_010697,NC_010699 |
