# Supplementary material for: MOCAT: A Metagenomics Assembly and Gene Prediction Toolkit
Source: PLoS One. 2012 Oct 17;7(10):e47656. doi: 10.1371/journal.pone.0047656 (PMC3474746; doi:10.1371/journal.pone.0047656)
Supplement: Table S4 — Reference sequences to which reads from the even HMP mock community were mapped. (DOCX) [file pone.0047656.s004.docx]

**Table S4.** Reference sequences to which reads from the even HMP mock community (http://www.hmpdacc.org/HMMC/) were mapped. All references were downloaded from NCBI, except *Candida albicans*, which was downloaded from http://www.candidagenome.org/download/sequence/C_albicans_SC5314/
Assembly21/current/.

| **Genome / Plasmid** | **GI** | **GB / REF** |
| --- | --- | --- |
| Acinetobacter baumannii ATCC 17978 | 126385999 | CP000521.1 |
| Acinetobacter baumannii ATCC 17978 plasmid pAB1 | 126640097 | NC_009083.1 |
| Acinetobacter baumannii ATCC 17978 plasmid pAB2 | 126640109 | NC_009084.1 |
| Actinomyces odontolyticus ATCC 17982 | 145666781 | AAYI02000004.1 |
| Bacillus cereus ATCC 10987 | 42740913 | AE017194.1 |
| Bacillus cereus ATCC 10987 plasmid pBc10987 | 44004339 | NC_005707.1 |
| Bacteroides vulgatus ATCC 8482 | 149931032 | CP000139.1 |
| Candida albicans SC5314, assembly 21 | - | SC5314 |
| Clostridium beijerinckii NCIMB 8052 | 149901357 | CP000721.1 |
| Deinococcus radiodurans R1 chromosome 1 | 15805042 | NC_001263.1 |
| Deinococcus radiodurans R1 chromosome 2 | 15807672 | NC_001264.1 |
| Deinococcus radiodurans R1 plasmid CP1 | 10957530 | NC_000959.1 |
| Deinococcus radiodurans R1 plasmid MP1 | 10957398 | NC_000958.1 |
| Enterococcus faecalis OG1RF | 327533853 | CP002621.1 |
| Escherichia coli str. K-12 substr. MG1655 | 48994873 | U00096.2 |
| Helicobacter pylori 26695 | 6626253 | AE000511.1 |
| Lactobacillus gasseri ATCC 33323 | 116628683 | NC_008530.1 |
| Listeria monocytogenes EGD-e | 16802048 | NC_003210.1 |
| Methanobrevibacter smithii ATCC 35061 | 148551077 | CP000678.1 |
| Neisseria meningitidis MC58 | 66731897 | AE002098.2 |
| Propionibacterium acnes KPA171202 | 50841496 | NC_006085.1 |
| Pseudomonas aeruginosa PAO1 | 110227054 | AE004091.2 |
| Rhodobacter sphaeroides 2.4.1 chromosome 1 | 77461965 | NC_007493.1 |
| Rhodobacter sphaeroides 2.4.1 chromosome 2 | 77464988 | NC_007494.1 |
| Rhodobacter sphaeroides 2.4.1 plasmid A | 125654605 | NC_009007.1 |
| Rhodobacter sphaeroides 2.4.1 plasmid B | 77404592 | NC_007488.1 |
| Rhodobacter sphaeroides 2.4.1 plasmid C | 77404693 | NC_007489.1 |
| Rhodobacter sphaeroides 2.4.1 plasmid D | 77404776 | NC_007490.1 |
| Rhodobacter sphaeroides 2.4.1 plasmid E | 125654693 | NC_009008.1 |
| Staphylococcus aureus subsp. aureus USA300_TCH1516 chromosome | 161508266 | NC_010079.1 |
| Staphylococcus aureus subsp. aureus USA300_TCH1516 plasmid pUSA300HOUMR | 161510924 | NC_010063.1 |
| Staphylococcus epidermidis ATCC 12228 chromosome | 27466918 | NC_004461.1 |
| Staphylococcus epidermidis ATCC 12228 plasmid pSE-122281 | 32470588 | NC_005008.1 |
| Staphylococcus epidermidis ATCC 12228 plasmid pSE-122282 | 32470581 | NC_005007.1 |
| Staphylococcus epidermidis ATCC 12228 plasmid pSE-122283 | 32470572 | NC_005006.1 |
| Staphylococcus epidermidis ATCC 12228 plasmid pSE-122284 | 32470555 | NC_005005.1 |
| Staphylococcus epidermidis ATCC 12228 plasmid pSE-122285 | 32470532 | NC_005004.1 |
| Staphylococcus epidermidis ATCC 12228 plasmid pSE-122286 | 32470520 | NC_005003.1 |
| Streptococcus agalactiae 2603V/R | 22535226 | AE009948.1 |
| Streptococcus mutans UA159 | 345287734 | AE014133.2 |
| Streptococcus pneumoniae TIGR4 | 193804931 | AE005672.3 |
