# Supplementary material for: MOCAT: A Metagenomics Assembly and Gene Prediction Toolkit
Source: PLoS One. 2012 Oct 17;7(10):e47656. doi: 10.1371/journal.pone.0047656 (PMC3474746; doi:10.1371/journal.pone.0047656)
Supplement: Table S5 — Aligned raw reads form the mock community to known Illumina adapters. (DOCX) [file pone.0047656.s005.docx]

**Table S5.** Aligned raw reads form the mock community to known Illumina adapters. These reads, in total 385,722 out of 6,562,065 (5.9%), were removed prior to assembly and mapping to reference sequences. Oligonucleotide sequences © 2006-2008 Illumina, Inc. All rights reserved.

| **Sequence** | **Reads matching sequence** |
| --- | --- |
| 5’-GATCGGAAGAGCTCGTATGCCGTCTTCTGCTTG-3’ | 229,378 |
| 5’-AGATCGGAAGAGCGGTTCAGCAGGAATGCCGAGACCG-3’ | 86,989 |
| 5’-CAAGCAGAAGACGGCATACGAGCTCTTCCGATC-3’ | 62,431 |
| 5’-AGATCGGAAGAGCGTCGTGTAGGGAAAGAGTGT-3’ | 4,776 |
| 5’-AGATCGGAAGAGCGTCGTGTAGGGAAAGAGTGT-3’ | 1,477 |
| 5’-AGATCGGAAGAGCGTCGTGTAGGGAAAGAGTGT-3’ | 1,056 |
| 5’-GATCGGAAGAGCACACGTCT-3’ | 213 |
| 5’-GATCGTCGGACTGTAGAACTCTGAAC-3’ | 159 |
| 5’-CAAGCAGAAGACGGCATACGA-3’ | 85 |
| 5’-AGATCGGAAGAGCGTCGTGTAGGGAAAGAGTGT-3’ | 46 |
| 5’-ACAGGTTCAGAGTTCTACAGTCCGACATG-3’ | 15 |
| 5’-ACAGGTTCAGAGTTCTACAGTCCGAC-3’ | 13 |
| 5’-AGACGTGTGCTCTTCCGATC-3’ | 9 |
| 5’-CATGTCGGACTGTAGAACTCTGAACCTGT-3’ | 8 |
| 5’-ACACTCTTTCCCTACACGACGCTCTTCCGATCT-3’ | 4 |
| 5’-CAAGCAGAAGACGGCATACGANN-3’ | 1 |
| 5’-GATCGGAAGAGCGGTTCAGCAGGAATGCCGAG-3’ | 1 |
| 5’-ACACTCTTTCCCTACACGACGCTCTTCCGATCT-3’ | 1 |
