# Supplementary material for: MOCAT: A Metagenomics Assembly and Gene Prediction Toolkit
Source: PLoS One. 2012 Oct 17;7(10):e47656. doi: 10.1371/journal.pone.0047656 (PMC3474746; doi:10.1371/journal.pone.0047656)
Supplement: Table S6 — Maximum computational resources and processing time required for each processing step, for each of the datasets used in this article. (DOC) [file pone.0047656.s006.doc]

**Table S6.** Maximum computational resources and processing time required for each processing step, for each of the datasets used in this article. For the 124 fecal metagenomes dataset [8], the largest sample (MH0012) has been used as an example. RAM indicates the required amount of RAM on the system, and HDD the amount of free hard disk space required. *In MOCAT this step is run in three separate steps (*screen, filter* and *calculate coverage*), and the database is dataset dependent. For MH0012, the database is the provided hg19 database.

| **Processing step** | **Mock Community** | **Simulated Dataset** | **MH0012** | **Time to process MH0012** |
| --- | --- | --- | --- | --- |
| Read Trim Filter | 70 MB RAM 1 MB HDD | 70 MB RAM  1 MB HDD | 70 MB RAM  1 MB HDD | 3 h 14 min  3 CPUs used |
| Adapter Screen | 160 MB RAM  1 GB HDD | - | 160 MB RAM  3.3 GB HDD | 1 h 14 min  2 CPUs used |
| Assembly | 4.6 GB RAM  100 MB HDD | 12 GB RAM  2.2 GB HDD | 54.4 GB RAM  14.2 GB HDD | 6 h 40 min  24 CPUs used |
| Assembly Revision | - | - | 28 GB RAM  48 GB HDD | 9 h 22 min  24 CPUs used |
| Gene Prediction | 130 MB RAM  1 MB HDD | 130 MB RAM  160 MB HDD | 100 MB RAM  1 MB HDD | 10 min  1 CPU used |
| Map reads to Database* | 2 GB RAM  1.2 GB HDD | 4.4 GB RAM  21 GB HDD | 5.9 GB RAM  11 GB HDD | 3 h 35 min  8 CPUs used |
| Total HDD space required (GB) | 2.5 | 26 | 56 | - |
| Size of sample (Gbp) | 0.5 | 4.0 | 14.0 | - |
