# Supplementary material for: MOCAT: A Metagenomics Assembly and Gene Prediction Toolkit
Source: PLoS One. 2012 Oct 17;7(10):e47656. doi: 10.1371/journal.pone.0047656 (PMC3474746; doi:10.1371/journal.pone.0047656)
Supplement: Table S7 — Output files and statistics from each of the processing steps in MOCAT. (DOCX) [file pone.0047656.s007.docx]

**Table S7.** Output files and statistics from each of the processing steps in MOCAT. Most of these statistics are generated after each processing step, but some statistics will be generated for a set of samples by executing MOCAT with the *–ss* option.

| **Processing step** | **Output files** | **Statistics** |
| --- | --- | --- |
| Read Trim Filter | Paired-end and single-end HQ reads | Number of HQ reads, bases and inserts; maximum and average read length, estimated kmer |
| Screen Fasta File | One set of paired-end and single-end reads files with reads matching the searched sequences; one set of files with reads not matching the sequences; original output and log files from Usearch; IDs of reads matching searched sequences | Number of reads, bases and inserts remaining after removal of reads matching sequences; re-estimated maximum and average read length; re-estimated kmer; Number of total and unique reads matching fasta sequences; Number of reads matching each individual fasta sequence |
| Screen Database | One set of paired-end and single-end reads files with reads matching the searched sequences; one set of files with reads not matching the sequences; SOAPaligner output file in SOAP and SAM format; IDs of reads matching searched database | Number of reads, bases and inserts remaining after removal of reads matching sequences; re-estimated maximum and average read length; re-estimated kmer |
| Assembly | Config file used to initiate SOAPdenovo; assembled contigs, scaffolds and scaftigs | Estimated insert size; N50, N90, total length, longest sequence and number of sequences for contigs, scaffolds and scaftigs longer than 100, 200, 500, 1000 bp; detailed number of sequences, GC content, nucleotide content, median, mean, maximum and minimum length of scaffolds and contigs |
| Assembly Revision | Revised scaftigs | Number of single base errors, number of small insertions, total length of insertions, number of small deletions, total length of deletions, number of chimeric-like regions, total length of chimeric-like regions |
| Gene Prediction | Protein and DNA sequences of genes; Information on each gene in tabular format; original output file from either MetaGeneMark or Prodigal | Contig ID of gene; gene start and stop; gene length; gene completeness; strand; total number of genes, complete genes and incomplete genes; Number of genes with start and stop codon; total gene length of all, complete and incomplete genes |
| Filter Screened Reads | BAM or SOAP formatted file with reads matching a database with a length and percentage identity above set thresholds | - |
| Calculate Coverage | Length normalized and non normalized base and read coverage | Total number of inserts and bases; Total number of mapped inserts and bases; fractions of mapped bases and inserts |
