# Supplementary material for: MOCAT: A Metagenomics Assembly and Gene Prediction Toolkit
Source: PLoS One. 2012 Oct 17;7(10):e47656. doi: 10.1371/journal.pone.0047656 (PMC3474746; doi:10.1371/journal.pone.0047656)
Supplement: Table S8 — Number of raw and high quality (HQ) bases and reads, calculated Kmer size, and the computational resources (RAM and HDD) required to assemble the 124 fecal metagenomics samples. (DOCX) [file pone.0047656.s008.docx]

**Table S8.** Number of raw and high quality (HQ) bases and reads, calculated Kmer size, and the computational resources (RAM and HDD) required to assemble the 124 fecal metagenomics samples.

| **Sample** | **Raw bases** | **HQ bases** | **Raw reads** | **HQ reads** | **Kmer** | **RAM (GB)** | **HDD (GB)** |
| --- | --- | --- | --- | --- | --- | --- | --- |
| MH0001 | 1,980,656,568 | 1,765,014,118 | 45,014,922 | 44,157,339 | 21 | 12.1 | 12 |
| MH0002 | 3,492,304,050 | 2,846,439,865 | 46,564,054 | 44,777,507 | 33 | 29.8 | 5.2 |
| MH0003 | 3,778,624,650 | 2,954,916,413 | 50,381,662 | 46,612,846 | 33 | 29.9 | 5.4 |
| MH0004 | 1,772,189,144 | 1,606,449,283 | 40,277,026 | 39,843,322 | 21 | 12.2 | 2.2 |
| MH0005 | 1,733,708,328 | 1,582,603,563 | 39,402,462 | 38,902,849 | 21 | 12.1 | 2.2 |
| MH0006 | 10,901,069,708 | 9,135,943,817 | 161,145,974 | 154,961,666 | 31 | 36.8 | 12.4 |
| MH0007 | 1,625,848,136 | 1,486,250,301 | 36,951,094 | 36,654,059 | 21 | 12.1 | 2.1 |
| MH0008 | 1,678,787,264 | 1,530,772,431 | 38,154,256 | 37,718,859 | 21 | 12.1 | 2.1 |
| MH0009 | 4,383,459,900 | 2,415,703,894 | 58,446,132 | 39,735,195 | 31 | 20.1 | 4.8 |
| MH0010 | 1,723,349,320 | 1,579,123,550 | 39,167,030 | 38,765,487 | 21 | 12 | 2.3 |
| MH0011 | 4,386,955,200 | 3,067,928,844 | 58,492,736 | 49,865,051 | 33 | 29.8 | 5.5 |
| MH0012 | 13,953,809,850 | 11,021,022,013 | 186,050,798 | 171,790,727 | 33 | 54.4 | 14.2 |
| MH0013 | 1,760,197,296 | 1,591,559,249 | 40,004,484 | 39,757,339 | 21 | 12 | 2.2 |
| MH0014 | 4,222,174,200 | 2,873,400,376 | 56,295,656 | 45,081,880 | 33 | 29.8 | 5.2 |
| MH0015 | 1,452,565,400 | 1,287,850,754 | 33,012,850 | 32,119,623 | 21 | 12.1 | 1.8 |
| MH0016 | 4,079,361,450 | 2,451,897,896 | 54,391,486 | 39,979,593 | 31 | 12.1 | 4.5 |
| MH0017 | 1,590,717,392 | 1,422,365,742 | 36,152,668 | 35,093,117 | 21 | 12 | 2 |
| MH0018 | 1,609,412,904 | 1,421,216,976 | 36,577,566 | 35,323,995 | 21 | 7.9 | 2.1 |
| MH0019 | 1,709,575,384 | 1,369,107,011 | 38,853,986 | 35,160,036 | 21 | 12.1 | 1.8 |
| MH0020 | 3,434,593,200 | 2,001,557,772 | 45,794,576 | 33,709,388 | 31 | 12 | 4.8 |
| MH0021 | 1,969,058,700 | 1,653,416,123 | 26,254,116 | 25,701,034 | 33 | 17.6 | 3.7 |
| MH0022 | 1,632,769,952 | 1,269,655,610 | 37,108,408 | 33,205,430 | 21 | 12.1 | 1.7 |
| MH0023 | 1,662,359,248 | 1,408,676,988 | 37,780,892 | 35,875,748 | 21 | 12 | 1.9 |
| MH0024 | 3,615,740,280 | 1,773,710,279 | 54,906,872 | 30,106,247 | 31 | 12 | 4.6 |
| MH0025 | 3,678,992,940 | 2,058,012,585 | 55,597,758 | 33,850,486 | 31 | 20.3 | 5.1 |
| MH0026 | 2,811,106,650 | 2,027,675,539 | 37,481,422 | 35,524,547 | 29 | 12 | 4.4 |
| MH0027 | 1,424,516,016 | 1,271,117,803 | 32,375,364 | 31,222,855 | 21 | 12.1 | 1.8 |
| MH0028 | 3,618,371,490 | 2,238,168,862 | 55,147,450 | 36,743,769 | 31 | 20.2 | 5.4 |
| MH0030 | 3,399,735,120 | 1,680,771,539 | 51,598,890 | 30,492,533 | 29 | 12 | 4.2 |
| MH0031 | 3,653,785,380 | 1,734,168,139 | 55,512,226 | 29,771,863 | 31 | 12 | 4.7 |
| MH0032 | 3,279,648,690 | 1,833,086,109 | 50,094,084 | 32,519,548 | 29 | 11.9 | 4.4 |
| MH0033 | 3,374,502,600 | 1,863,210,985 | 44,993,368 | 32,274,975 | 31 | 12 | 4.3 |
| MH0034 | 2,804,976,750 | 1,461,413,009 | 37,399,690 | 24,297,412 | 31 | 12 | 4.4 |
| MH0035 | 3,694,199,550 | 2,031,770,898 | 49,255,994 | 34,448,891 | 31 | 12.1 | 5 |
| MH0036 | 3,577,563,150 | 1,828,007,006 | 47,700,842 | 34,749,300 | 27 | 11.9 | 4.1 |
| MH0037 | 3,152,310,900 | 2,000,734,854 | 42,030,812 | 36,799,294 | 29 | 11.9 | 4.7 |
| MH0038 | 3,409,432,050 | 1,733,182,713 | 45,459,094 | 32,506,123 | 27 | 12 | 3.8 |
| MH0039 | 3,203,633,550 | 1,951,571,591 | 42,715,114 | 35,595,822 | 29 | 12 | 4.6 |
| MH0040 | 3,318,569,850 | 2,037,478,379 | 44,247,598 | 32,396,033 | 33 | 29.8 | 4.7 |
| MH0041 | 3,327,706,350 | 2,394,709,932 | 44,369,418 | 37,556,157 | 33 | 17.5 | 5.2 |
| MH0042 | 3,255,474,600 | 1,563,992,395 | 43,406,328 | 28,310,845 | 29 | 11.9 | 3.4 |
| MH0043 | 3,700,256,250 | 2,142,069,646 | 49,336,750 | 36,506,430 | 31 | 12 | 5.2 |
| MH0044 | 3,364,640,400 | 2,010,280,681 | 44,861,872 | 32,864,867 | 31 | 12.1 | 4.8 |
| MH0045 | 3,707,204,400 | 2,093,586,749 | 49,429,392 | 35,780,144 | 31 | 12 | 5.1 |
| MH0046 | 2,684,557,950 | 1,510,183,429 | 35,794,106 | 27,551,388 | 29 | 12 | 3.6 |
| MH0047 | 2,019,686,100 | 1,694,808,747 | 26,929,148 | 26,602,136 | 33 | 17.5 | 4.3 |
| MH0048 | 2,022,570,300 | 1,353,735,846 | 26,967,604 | 23,544,675 | 29 | 12 | 3.2 |
| MH0049 | 2,298,981,900 | 1,882,322,065 | 30,653,092 | 30,072,977 | 33 | 17.5 | 4.4 |
| MH0050 | 3,584,220,750 | 2,330,112,791 | 47,789,610 | 41,300,537 | 29 | 20.2 | 5.2 |
| MH0051 | 1,946,598,300 | 1,373,577,424 | 25,954,644 | 23,345,697 | 31 | 12 | 3.4 |
| MH0052 | 2,142,943,950 | 1,910,794,842 | 28,572,586 | 28,129,666 | 35 | 29.7 | 4.7 |
| MH0053 | 3,246,061,800 | 2,314,408,702 | 43,280,824 | 38,798,868 | 31 | 20.2 | 4.9 |
| MH0054 | 3,169,381,350 | 2,217,323,804 | 42,258,418 | 37,293,736 | 31 | 20.2 | 4.8 |
| MH0055 | 3,533,101,950 | 2,192,467,255 | 47,108,026 | 38,116,708 | 31 | 11.9 | 5.1 |
| MH0056 | 3,685,113,000 | 1,782,244,203 | 49,134,840 | 32,560,651 | 29 | 20.3 | 4 |
| MH0057 | 3,228,504,900 | 1,944,409,715 | 43,046,732 | 32,693,045 | 31 | 12 | 4.4 |
| MH0058 | 3,735,938,700 | 2,251,421,570 | 49,812,516 | 39,784,242 | 29 | 20.2 | 4.7 |
| MH0059 | 3,076,077,600 | 2,270,727,318 | 41,014,368 | 36,977,037 | 31 | 20.2 | 5.3 |
| MH0060 | 3,083,246,400 | 2,369,447,024 | 41,109,952 | 38,541,390 | 31 | 20.2 | 5.1 |
| MH0061 | 3,333,300,300 | 1,941,349,510 | 44,444,004 | 30,679,299 | 33 | 17.6 | 5.1 |
| MH0062 | 2,738,421,900 | 2,144,293,717 | 36,512,292 | 34,658,824 | 33 | 17.6 | 4.7 |
| MH0063 | 3,446,813,850 | 2,016,936,340 | 45,957,518 | 33,086,603 | 31 | 20.2 | 5.1 |
| MH0064 | 3,354,583,650 | 1,941,820,225 | 44,727,782 | 31,567,330 | 33 | 17.6 | 5.1 |
| MH0065 | 3,343,047,300 | 1,679,984,833 | 44,573,964 | 27,883,351 | 31 | 11.9 | 4.7 |
| MH0066 | 2,708,468,250 | 1,828,423,401 | 36,112,910 | 31,008,724 | 31 | 11.9 | 4.7 |
| MH0067 | 3,336,258,300 | 2,026,207,336 | 44,483,444 | 32,057,365 | 33 | 17.5 | 5.1 |
| MH0068 | 3,289,801,200 | 2,063,332,916 | 43,864,016 | 34,185,667 | 31 | 12.1 | 5.1 |
| MH0069 | 5,228,371,950 | 3,506,359,699 | 69,711,626 | 57,773,857 | 31 | 20.3 | 6.3 |
| MH0070 | 3,593,524,650 | 1,970,739,028 | 47,913,662 | 33,656,254 | 31 | 12 | 4.9 |
| MH0071 | 3,467,615,400 | 2,124,696,838 | 46,234,872 | 36,107,652 | 31 | 12 | 5.2 |
| MH0072 | 3,695,709,450 | 894,456,215 | 49,276,126 | 17,206,199 | 27 | 7.9 | 1.6 |
| MH0073 | 3,542,143,800 | 1,826,741,688 | 47,228,584 | 29,868,645 | 31 | 11.9 | 5 |
| MH0074 | 3,452,174,700 | 2,054,100,382 | 46,028,996 | 35,395,597 | 31 | 12 | 5 |
| MH0075 | 3,529,770,300 | 2,060,190,316 | 47,063,604 | 34,463,110 | 31 | 11.9 | 5.1 |
| MH0076 | 2,298,177,450 | 1,937,861,984 | 30,642,366 | 30,208,162 | 33 | 17.6 | 4.5 |
| MH0077 | 3,723,730,950 | 2,245,323,604 | 49,649,746 | 36,910,358 | 31 | 20.2 | 5.3 |
| MH0078 | 1,950,900,450 | 1,797,533,428 | 26,012,006 | 25,799,905 | 37 | 17.5 | 4.1 |
| MH0079 | 2,056,121,250 | 1,708,373,409 | 27,414,950 | 26,262,009 | 33 | 17.6 | 4.2 |
| MH0080 | 3,493,807,650 | 2,117,701,313 | 46,584,102 | 36,776,519 | 31 | 11.9 | 5 |
| MH0081 | 3,588,761,850 | 2,209,968,828 | 47,850,158 | 35,399,534 | 33 | 17.5 | 5.4 |
| MH0082 | 3,625,673,250 | 2,250,385,478 | 48,342,310 | 36,782,367 | 31 | 20.1 | 5.4 |
| MH0083 | 3,592,663,050 | 2,448,606,320 | 47,902,174 | 41,192,467 | 31 | 20.2 | 5.4 |
| MH0084 | 4,017,078,600 | 1,727,403,462 | 53,561,048 | 31,203,907 | 29 | 7.8 | 3.5 |
| MH0085 | 3,707,425,800 | 2,368,902,495 | 49,432,344 | 40,084,145 | 31 | 12 | 5.3 |
| MH0086 | 4,157,294,400 | 2,986,565,858 | 55,430,592 | 47,440,137 | 33 | 29.7 | 6.2 |
| O2.UC1-0 | 3,135,329,250 | 1,840,331,700 | 41,804,390 | 33,364,340 | 29 | 12 | 4.6 |
| O2.UC11-0 | 2,887,532,400 | 1,540,390,566 | 38,500,432 | 24,993,670 | 33 | 17.5 | 4.4 |
| O2.UC12-0 | 2,767,796,700 | 1,491,736,430 | 36,903,956 | 22,602,619 | 35 | 17.5 | 4.2 |
| O2.UC13-0 | 3,271,212,450 | 1,544,219,476 | 43,616,166 | 27,873,117 | 29 | 11.9 | 4.4 |
| O2.UC14-0 | 2,020,545,900 | 823,606,428 | 26,940,612 | 12,557,394 | 35 | 11.4 | 1.9 |
| O2.UC16-0 | 3,170,095,350 | 1,544,995,579 | 42,267,938 | 28,404,209 | 29 | 11.9 | 4.4 |
| O2.UC17-0 | 3,022,717,350 | 1,640,759,832 | 40,302,898 | 28,960,255 | 29 | 12 | 4.5 |
| O2.UC18-0 | 3,142,523,850 | 1,754,884,805 | 41,900,318 | 29,620,491 | 31 | 7.7 | 4.3 |
| O2.UC19-0 | 2,884,363,800 | 1,694,124,338 | 38,458,184 | 27,334,094 | 33 | 17.6 | 4.1 |
| O2.UC20-0 | 2,878,088,850 | 1,686,017,942 | 38,374,518 | 27,414,586 | 33 | 17.4 | 4.1 |
| O2.UC21-0 | 2,611,804,950 | 726,382,734 | 34,824,066 | 13,854,655 | 27 | 7.8 | 1.7 |
| O2.UC22-0 | 3,318,153,000 | 1,731,978,642 | 44,242,040 | 31,447,773 | 29 | 12 | 4.5 |
| O2.UC23-0 | 2,618,447,550 | 1,522,181,878 | 34,912,634 | 24,009,707 | 33 | 17.5 | 4 |
| O2.UC24-0 | 3,123,249,750 | 1,985,526,389 | 41,643,330 | 31,966,247 | 33 | 17.5 | 5 |
| O2.UC4-0 | 3,244,125,450 | 1,510,115,549 | 43,255,006 | 27,987,260 | 29 | 12 | 4.4 |
| V1.CD1-0 | 3,534,363,600 | 2,042,114,835 | 47,124,848 | 34,764,418 | 31 | 12 | 4.3 |
| V1.CD11-0 | 3,854,072,550 | 1,191,678,518 | 51,387,634 | 21,643,298 | 29 | 7.8 | 2 |
| V1.CD12-0 | 3,045,568,050 | 2,272,910,242 | 40,607,574 | 35,853,514 | 33 | 17.5 | 4.7 |
| V1.CD13-0 | 3,191,908,200 | 2,338,179,144 | 42,558,776 | 38,285,234 | 31 | 20.2 | 5.2 |
| V1.CD14-0 | 3,301,533,300 | 2,511,787,382 | 44,020,444 | 39,787,419 | 33 | 29.9 | 5.6 |
| V1.CD15-0 | 3,035,162,550 | 2,499,672,479 | 40,468,834 | 39,178,785 | 33 | 17.4 | 5.2 |
| V1.CD2-0 | 3,501,036,000 | 1,966,728,530 | 46,680,480 | 34,148,871 | 31 | 12 | 4.2 |
| V1.CD3-0 | 3,400,291,200 | 1,993,843,777 | 45,337,216 | 33,570,149 | 31 | 12 | 4.4 |
| V1.CD4-0 | 3,220,252,050 | 1,771,708,149 | 42,936,694 | 29,313,325 | 31 | 12 | 4.2 |
| V1.CD6-0 | 3,177,562,500 | 1,630,352,518 | 42,367,500 | 27,347,478 | 31 | 12 | 3.9 |
| V1.CD8-0 | 3,742,693,650 | 1,750,830,334 | 49,902,582 | 31,557,705 | 29 | 11.9 | 3.5 |
| V1.CD9-0 | 3,534,963,300 | 1,887,983,777 | 47,132,844 | 32,399,692 | 31 | 12 | 3.9 |
| V1.UC10-0 | 3,094,771,200 | 2,026,650,930 | 41,263,616 | 35,379,849 | 29 | 20.2 | 4.9 |
| V1.UC13-0 | 2,862,227,700 | 2,091,082,884 | 38,163,036 | 36,042,895 | 31 | 11.9 | 4.6 |
| V1.UC14-0 | 3,718,260,450 | 2,140,460,385 | 49,576,806 | 40,341,131 | 27 | 12 | 5 |
| V1.UC15-0 | 3,486,056,550 | 1,810,309,407 | 46,480,754 | 35,441,830 | 27 | 12 | 4.1 |
| V1.UC17-0 | 2,501,392,050 | 1,022,705,084 | 33,351,894 | 19,602,665 | 27 | 12 | 2.3 |
| V1.UC18-0 | 3,627,965,400 | 1,972,813,409 | 48,372,872 | 36,904,157 | 27 | 11.9 | 4.9 |
| V1.UC19-0 | 3,692,097,900 | 2,086,334,121 | 49,227,972 | 38,905,989 | 29 | 11.9 | 5 |
| V1.UC21-0 | 3,473,233,050 | 1,808,207,802 | 46,309,774 | 31,770,781 | 29 | 12 | 4.7 |
| V1.UC6-0 | 3,235,557,600 | 2,554,804,294 | 43,140,768 | 41,190,295 | 33 | 29.9 | 5.5 |
| V1.UC7-0 | 2,738,716,650 | 2,232,646,259 | 36,516,222 | 35,210,767 | 33 | 17.5 | 4.8 |
| V1.UC8-0 | 3,498,453,750 | 2,470,786,099 | 46,646,050 | 43,591,898 | 29 | 20.2 | 5.3 |
| V1.UC9-0 | 3,517,717,350 | 2,284,987,896 | 46,902,898 | 41,256,192 | 29 | 12 | 5.1 |
